# Genome-Wide Association Study Reveals the Genetic Architecture of Stripe Rust Resistance at the Adult Plant Stage in Chinese Endemic Wheat

**DOI:** 10.3389/fpls.2020.00625

**Published:** 2020-06-05

**Authors:** Jing Li, Yunfeng Jiang, Fangjie Yao, Li Long, Yuqi Wang, Yu Wu, Hao Li, Jirui Wang, Qiantao Jiang, Houyang Kang, Wei Li, Pengfei Qi, Jian Ma, Zhien Pu, Shoufen Dai, Yuming Wei, Youliang Zheng, Guoyue Chen

**Affiliations:** ^1^Triticeae Research Institute, Sichuan Agricultural University, Chengdu, China; ^2^State Key Laboratory of Crop Gene Exploitation and Utilization in Southwest China, Chengdu, China; ^3^College of Agronomy, Sichuan Agricultural University, Chengdu, China

**Keywords:** Chinese endemic wheat, stripe rust, GWAS, Tibetan semi-wild wheat, Yunnan hulled wheat, Xinjiang rice wheat

## Abstract

Chinese endemic wheat, comprising Tibetan semi-wild wheat (*Triticum aestivum* ssp. *tibetanum*), Yunnan hulled wheat (*T. aestivum* ssp. *yunnanense*), and Xinjiang rice wheat (*T. petropavlovskyi*), are genetically and morphologically unique. To examine the adult plant resistance to stripe rust among Chinese endemic wheat germplasms, a panel of 213 accessions was inoculated with mixed virulent races of wheat stripe rust (*Puccinia striiformis* f. sp. *tritici*) in four different field environments. Four traits associated with stripe rust resistance, infection type, final disease severity, disease index, and area under the disease progress curve, were used to evaluate the accessions. The phenotypic datasets were used for 55K single-nucleotide polymorphism (SNP) array-based genome-wide association studies to identify effective resistance loci. Eighty-nine accessions with stable resistance were identified in at least three of the four environments by phenotypic evaluation. Eleven markers located on chromosomes 1A, 2B, 5A, 5D, 7B, and 7D by the genome-wide association studies analysis showed significant associations with at least two resistance-associated traits in two of the environments. These loci, corresponding to seven genomic regions based on linkage disequilibrium decay distance, explained 9.3 to 26.0% of the total phenotypic variation. Five quantitative trait loci (QTLs) on chromosomes 1A, 2B, 7B, and 7D overlapped or were in close proximity to previously reported QTLs based on the consensus and physical maps using the reference sequence of bread wheat (IWGSC RefSeq v1.0). The other two QTLs were potential novel QTLs given their physical positions. Haplotype variants of QTL *QYr.sicau-2BS* showed subspecies-specific inheritance of the stripe rust resistance locus. Resistant loci among Chinese endemic wheat germplasms could be introduced into common wheat cultivars, and the high-confidence SNP markers will aid in marker-assisted selection in breeding for stripe rust disease resistance.

## Introduction

Bread wheat (*Triticum aestivum* L.; genomes AABBDD, 2*n* = 42) is a hexaploid wheat and a leading cereal crop grown worldwide that provides 20% of the global caloric requirements (Shewry and Hey, 2015). Eight subspecies of hexaploid wheat harbor many beneficial alleles with potential for bread wheat improvement. They are Club wheat (*T. aestivum* ssp. *compactum*), Indian dwarf wheat (*T. aestivum* ssp. *sphaerococcum*), Spelt wheat (*T. aestivum* ssp. *spelta*), Macha wheat (*T. aestivum* ssp. *macha*), Vavilovii wheat (*T. aestivum* ssp. *vavilovii*), Tibetan semi-wild wheat (*T. aestivum* ssp. *tibetanum* Shao), Yunnan hulled wheat (*T. aestivum* ssp. *yunnanense*), and Xinjiang rice wheat (*T. petropavlovskyi* Udacz et. Migusch) (Dorofeev et al., 1979; Dong and Zheng, 1999; Mac Key, 2005). Among them, Tibetan semi-wild wheat, Yunnan hulled wheat, and Xinjiang rice wheat are unique to China and constitute Chinese endemic wheat (Dong and Zheng, 1999).

Tibetan semi-wild wheat is a weedy wheat that is naturally distributed only on the Qinghai-Tibet Plateau of China. It is grown mainly at altitudes above 3,000 m and coexists with wheat cultivars or landraces (Yang et al., 1993). It was first collected by Shao and his group in 1974 and identified as a subspecies of hexaploid wheat (Shao et al., 1980). Tibetan semi-wild wheat is characterized by spontaneous spike disarticulation, a tough glume, and seed dormancy, which indicates that it is closer to wild wheat species than other extant hexaploid wheats (Shao et al., 1980). Tibetan semi-wild wheat originated by a unique “de-domestication” process from hexaploid bread wheat (Jiang et al., 2019). Yunnan hulled wheat is grown mainly at altitudes between 1,500 and 2,500 m in the mountainous region of Yunnan province. It was first collected by Jin and his group in 1937 (Dong et al., 1981). It has a tough glume and brittle rachis, but differs considerably from Tibetan semi-wild wheat, spelt wheat and Macha wheat (Dong et al., 1981). Xinjiang rice wheat, also known as “Daosuimai” or rice-head wheat, was discovered in the Talimu basin of Xinjiang Province in China. Its most typical characteristic is the spike, which is similar to that of Polish wheat (*T. polonicum* L.) (Yao et al., 1983). Xinjiang rice wheat was classified initially as a tetraploid, but was recognized as a hexaploid after cytological examination by Udachin and Migushova in 1970 (Yang et al., 1993; Dong and Zheng, 1999).

Chinese endemic wheat evolved from primitive hexaploid wheat by natural and artificial selection under various ecological environments in China (Dong et al., 1981). Abundant genetic diversity has been discovered in studies of their morphology (Shao et al., 1980; Chen, 1980; Dong et al., 1981; Huang et al., 2002), agronomic traits (Wang et al., 2010), high-molecular-weight glutenin subunits (Wei et al., 2002; Wang et al., 2005a), *Gli-1*, *Gli-2*, and *Glu-1* alleles (Wei et al., 2001; Wang et al., 2005b), biochemistry, and molecular markers (Ward et al., 1998; Huang et al., 2002; Wang et al., 2007). Additionally, these subspecies exhibit several traits of biological and economic significance, such as stress tolerance (Guo et al., 2001), disease-resistance (Yang et al., 2011, 2012), and good quality (Cui and Ma, 1988). Given their high crossability with bread wheat, Chinese endemic wheat subspecies are considered valuable components of the primary gene pool useful for broadening the genetic base of germplasm in breeding programs.

Wheat stripe rust, which is caused by *Puccinia striiformis* f. sp. *tritici* (*Pst*), is a severely destructive disease of wheat worldwide. Identification of novel stripe-rust resistance genes and cultivation of resistant cultivars are considered the most effective approaches to control this disease (Chen, 2014). Chinese endemic wheat has been historically underused in breeding programs, and thus represent potential new sources of novel resistance alleles. To date, few studies have systematically identified and evaluated stripe rust resistance among Chinese endemic wheat germplasms (Li and Wu, 2005; Yang et al., 2012). Genome-wide association study (GWAS), a strategy to mine genetic variation affecting complex traits by correlation analysis, has been successfully applied to explore stripe rust resistance loci in wheat (Maccaferri et al., 2015; Bulli et al., 2016; Juliana et al., 2019). In the present study, a panel of 213 Chinese endemic wheat accessions (117 Tibetan semi-wild wheat, 78 Yunnan hulled wheat, and 18 Xinjiang rice wheat) was evaluated for resistance against *Pst* at the adult plant stage in multiple years and field locations. The objectives were to assess the genetic diversity, population structure, and linkage disequilibrium (LD) patterns of the accessions using 55K single-nucleotide polymorphism (SNP) array-based markers, to identify stripe rust resistant accessions, and to explore the genetic architecture of stripe rust resistance loci by GWAS analysis.

## Materials and Methods

### Plant Materials

We collected 213 Chinese endemic wheat accessions from the Triticeae Germplasm Resources Bank of Sichuan Agricultural University and the Chinese Crop Germplasm Resources Bank. Among these accessions, 117 were Tibetan semi-wild wheat, 78 were Yunnan hulled wheat, and 18 were Xinjiang rice wheat (Supplementary Table S1).

### Disease Phenotyping in Four Environments

To identify and evaluate the responses of these accessions to wheat stripe rust, we planted them in four environments in Sichuan Province: Chongzhou (30°33′N, 103°39′E) in 2018 and 2019 (one trial in 2018 and two trials in 2019), and Wenjiang (30°43′N, 103°52′E) in 2019, hereafter referred to as 18CZ, 19CZ1, 19CZ2, and 19WJ, respectively. The accessions were planted in three non-replicated rows in each environment. The rows were 2 m long and spaced 0.3 m apart, and 20 seeds of each accession were sown with 0.1 m inter-plant spacing. Seeds of the bread wheat cultivar “Avocet S” were sown every 20 rows as susceptible checks, and seeds of “SY95-71” were sown around the experimental fields as spreader rows. Seven virulent races of *Pst* prevalent in China (CYR32, CYR33, CYR34, Su11-4, Su11-5, Su11-7, and G22-14) were mixed with dry talc for smear inoculation at the tillering stage (Carter et al., 2009). The avirulence/virulence formula of the seven races was summarized in Supplementary Table S2.

Three random individual plants of each accession from each row-plots were marked with signboards to implement the biological duplications. The individuals in marginal areas were excluded to avoid the marginal effect. Disease severity (DS) was measured as the percentage infected leaf area. It was recorded three times in 7-day intervals for three random individual plants from the time when the DS values of the major susceptible checks attained 70–80% (Roelfs, 1992). The average final DS of three random individual plants was used in the phenotypic and GWAS analyses. Average disease incidence (I) was calculated as the number of diseased leaves/total leaves of the three random individual plants. Infection type (IT) was recorded at the last evaluation using the Stakman scale (0, 1, 2, 3, and 4) of Stakman et al. (1962), which we converted to 1, 2, 3, 4, 5, and 6, prior to the statistical analysis. IT values of 1–4 were considered to indicate resistance and 5–6 were considered to indicate susceptible (Yao et al., 2019). The average final DS of each accession was used to calculate the disease index (DI) as DI = I × DS (Zeng and Luo, 2006). The area under the disease progress curve (AUDPC) was calculated using the formula AUDPC = $\sum_{i=1}^{n-1}[(x_{i+1}+x_{i}/2\left(t_{i+1}-t_{i}\right)$, where *x*_*i*_, is the flag leaf rust severity on the *i*th date, *t*_*i*_ is the *i*th day, and *n* is number of times DS was recorded (Das et al., 1992). The DI and AUDPC used to indicate the disease progression for each accession.

### Phenotypic Data Analysis

Variance and correlation analyses of all traits (DS, IT, DI, and AUDPC) were computed using the “cor” package in R using the Pearson’s correlation method (Pandey et al., 2015). The R package “lme4” was used to calculate the best linear unbiased prediction (BLUP) values of each trait with a linear model and random effects for variance components to eliminate the impact of variation between environments on stripe rust (Bates et al., 2014). The broad-sense heritability (*H*^2^) estimates for each trait across the four environments were calculated as *H*^2^ = *V*_G_/(*V*_G_ + *V*_E_), where *V*_G_ represents genotypic variance and *V*_E_ represents environmental variance (Smith et al., 1998). The genotypic and environmental variances were obtained from the BLUP calculations. The highest and lowest values for each of the traits in each environment as well as the BLUP were determined. The mean, standard deviation (STDEV), and coefficient of variation (CV) also were calculated for each of the traits.

### Genotyping and Genetic Analysis

Leaf tissues were collected from 10 2-week-old seedlings for genomic DNA extraction using the modified cetyltrimethyl-ammonium bromide method (Saghaimaroof et al., 1984). Genotyping of the 213 accessions was conducted by CapitalBio Technology (Beijing, China) using the wheat 55K SNP array (Affymetrix^®^ Axiom^®^ Wheat55K). Markers with missing values ≤5% and minor allele frequencies ≥5% were selected for the subsequent genetic analysis.

Gene diversity, polymorphism information content (PIC), and major allele frequency (MAF) were calculated using POWERMARKER v3.25 (Liu and Muse, 2005). A consensus neighbor-joining tree (NJ tree) was constructed using the neighbor-joining algorithm in MEGA X^1^ and visualized using the iTOL website^2^.

The population structure (*Q*-matrix) was analyzed with STRUCTURE v2.3.4 using a Bayesian clustering model (Hubisz et al., 2009). Using the admixture model, five independent runs were performed with *K*-values of 1–10, burn-in of 10,000 iterations, and Markov Chain Monte Carlo of 10,000 iterations. To determine the optimal *K* value, the web-based STRUCTURE HARVESTER was employed using the Δ*K* method (Earl and Vonholdt, 2012).

The kinship matrix (*K*-matrix) and the LD were calculated using TASSEL v5.2 (Bradbury et al., 2007). Heat maps were generated using the “pheatmap” R package v1.0.8 (Kolde, 2015) based on the *K*-matrix, and the LD decay plot was generated with the “ggplot2” R package (Ginestet, 2011) using *r*^2^ values >0.1 and distance between markers (pDiseq) <0.001.

The analysis of molecular variance (AMOVA) was performed using GeneAlEx 6.5 (Peakall and Smouse, 2006). On each chromosome, the markers having a PIC value from 0.30 to 0.35 were selected and a total of 5,646 SNPs were used for AMOVA (Eltaher et al., 2018). The number of subpopulations determined based on STRUCTURE results were used for AMOVA.

### Genome-Wide Association Analysis

Genome-wide association study analyses for resistance were performed using a mixed linear model with *K* and *Q* matrices as covariates in TASSEL v5.2 because both the model-based Bayesian and distance-based hierarchical clustering algorithms revealed a strong population structure in the panel. The phenotypic datasets of four traits (IT, DS, DI, and AUDPC) from five trials (four environments and BLUP values) and the genotypes of the 213 accessions were used in the calculation (Bradbury et al., 2007; Zhang et al., 2010). A Manhattan plot was generated with the R package “CMplot” to show the association results^3^. Loci, which are associated with at least two traits in at least three of the trials, with *P* < 0.0001, were considered as confident marker–trait associations. A false discovery rate (FDR) set as 10% was used to determine the *P*-value thresholds. The quantitative trait loci (QTLs) from the GWAS analysis were mapped using the R package “MarkerGWAS” coded by Wheatomics^4^. The comparisons between *Yr* genes or QTLs reported previously and associated QTLs identified in this study were performed based on the consensus map (Wang and Chen, 2017; Cheng et al., 2019) and physical map using the reference sequence of bread wheat^5^ (IWGSC RefSeq v1.0).

### Haplotype Analysis

Haplotype analysis was performed as described by Dinglasan et al. (2019). Haplotype networks were generated from QTLs with significant association and markers in strong LD using Haploview (Barrett et al., 2004). The TCS statistical parsimony network approach implemented in PopART (Leigh and Bryant, 2015) was used to estimate genealogies among common allelic variants. Colors consistent with the average disease score represented the respective haplotype groups and each subspecies. Statistical data were presented on the topographical map to show the frequency and distribution of haplotypes in each subspecies.

## Results

### Disease Responses to Stripe Rust at Adult Plant Stage

Four traits associated with stripe rust resistance (IT, DS, DI, and AUDPC) recorded in field trials in four environments were used to evaluate stripe rust resistance in 213 Chinese endemic wheat accessions (Supplementary Table S1). The results showed that 43, 103, 122, and 106 accessions exhibited resistance to stripe rust (IT ≤ 4) in 18CZ, 19CZ1, 19CZ2, and 19WJ, respectively. About 42% (89) of the accessions displayed stable resistance in at least three environments, which indicated that the Chinese endemic wheat panel included a relatively high proportion of resistant accessions. No significant difference was detected among environments because highly significant correlations (*P* < 0.001) were detected among all four field trials (Supplementary Table S3). Similar high correlations were detected among the IT, DS, DI, and AUDPC traits (Supplementary Figure S1). The heritability (*H*^2^) values for IT, DS, DI, and AUDPC were 0.83, 0.84, 0.81, and 0.77, respectively (Table 1).

**TABLE 1:** Summary of the stripe rust response between three subspecies at the adult plant stage.

Traits	Trials	Max			Min			Mean			Heritability
		Tibetan^a^	Yunnan^a^	Xinjiang^a^	Tibetan	Yunnan	Xinjiang	Tibetan	Yunnan	Xinjiang	
IT	18CZ	6	4	6	2	2	2	5.72	2.47	4.76	0.83
	19CZ1	6	4	6	3	1	4	5.08	2.67	4.59	
	19CZ2	6	4	6	3	2	3	4.76	2.65	4.06	
	19WJ	6	3	6	2	1	3	5.33	2.65	4.44	
	BLUP value	5.91	3.54	5.91	2.58	2.23	3.30	5.18	2.79	4.43	
DS	18CZ	100.00	10.00	100.00	0.00	0.00	0.00	63.62	1.35	43.14	0.84
	19CZ1	100.00	11.67	100.00	0.00	0.00	5.00	64.99	1.79	46.67	
	19CZ2	93.33	13.33	100.00	0.00	0.00	1.67	54.05	1.33	33.52	
	19WJ	100.00	1.67	100.00	0.00	0.00	0.00	79.10	0.09	40.56	
	BLUP value	95.70	10.37	97.29	3.39	1.80	4.98	64.36	3.21	39.92	
DI	18CZ	73.33	7.22	75.56	0.00	0.00	0.32	35.32	0.58	23.72	0.81
	19CZ1	80.00	5.57	93.33	0.31	0.00	2.73	43.30	0.76	37.31	
	19CZ2	80.00	5.20	86.67	0.00	0.00	0.14	33.31	0.64	23.37	
	19WJ	62.78	2.50	48.26	0.00	0.00	0.00	35.78	0.18	16.17	
	BLUP value	60.08	5.35	64.02	1.51	0.69	2.69	36.16	1.74	24.36	
AUDPC	18CZ	12.90	1.45	13.10	0.00	0.00	0.05	6.08	0.10	4.15	0.77
	19CZ1	10.00	0.63	11.40	0.04	0.00	0.34	5.26	0.09	4.56	
	19CZ2	8.77	0.63	9.80	0.00	0.00	0.01	3.61	0.07	2.56	
	19WJ	10.29	0.56	7.23	0.00	0.00	0.00	4.97	0.03	2.20	
	BLUP value	8.43	1.02	8.38	0.24	0.21	0.39	4.85	0.47	3.27	

*^a^Tibetan, Tibetan semi-wild wheat; Yunnan, Yunnan hulled wheat; Xinjiang, Xinjiang rice wheat. IT, infection type; DS, final disease severity; DI, disease index; AUDPC, area under disease progress curve.*

**FIGURE 1:**
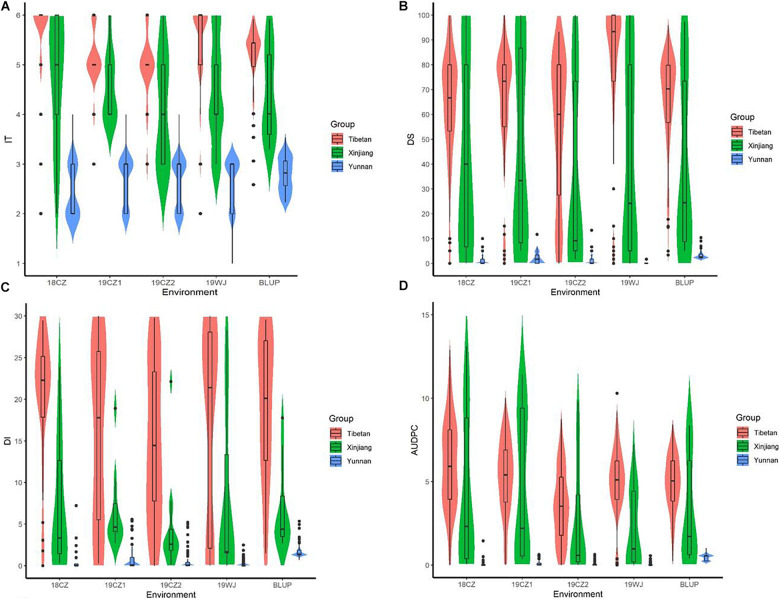
Box plots and violin plots of **(A)** infection type (IT), **(B)** final disease severity (DS), **(C)** disease index (DI) and **(D)** the area under disease progress curve (AUDPC) in five trials of three subspecies at the adult plant stage. Tests in Chongzhou from the year 2018 to 2019 was referred to as 18CZ, 19CZ1, and 19CZ2; in Wenjiang at the year 2019 was referred to as 19WJ. BLUP represented the best linear unbiased prediction value. Group of Tibetan represented Tibetan semi-wild wheat; Yunnan represented Yunnan hulled wheat; Xinjiang represented Xinjiang rice wheat. Solid horizontal lines showed the medians.

Significant differences in stripe rust response were observed among accessions from the three subspecies (Figure 1, Table 1). The Tibetan semi-wild wheat accessions predominantly exhibited highly susceptibility (>96% had ITs 5–6), and only four accessions (<4%) displayed stable resistance in three of the environments. The mean IT and DS values of the BLUP were 5.18 and 64.36%, respectively. Conversely, all 78 Yunnan hulled wheat accessions exhibited a high resistance (IT ≤ 3), with a mean IT of 2.79 and mean DS of 3.21% of the BLUP. About 39% (7) of the Xinjiang rice wheat accessions showed stable resistance and 61% (11) showed susceptibility. The mean IT of this panel in the BLUP was 4.43 and mean DS was 39.92%. The degree of resistance to stripe rust in the Chinese endemic wheat panel at the adult plant stage showed the trend Yunnan hulled wheat > Xinjiang rice wheat > Tibetan semi-wild wheat.

### Genetic Diversity Analysis

After filtering markers, a total of high-quality informative 38,490 polymorphic markers were used for genetic analysis. Among them, 14,564, 14,099, and 9,827 markers were located in the A, B, and D sub-genomes, with average densities of 3.0, 2.7, and 2.5 markers per Mb, respectively. Chromosome 2A carried the highest number of markers (2,347), whereas chromosome 1A had the highest marker density at 3.5 markers per Mb. Chromosome 4D carried the fewest markers (667) and had the lowest marker density at 1.3 markers per Mb. Gene diversity, PIC, and MAF for the chromosomes were 0.202–0.333, 0.176–0.283, and 0.753–0.867, respectively. Subgenome D had the highest MAF (0.832) and subgenome B had the highest gene diversity (0.285) and PIC (0.263). Chromosome 2D had the highest MAF (0.867), lowest genetic diversity (0.202), and lowest PIC (0.176), and chromosome 6A had the lowest MAF (0.753), highest genetic diversity (0.333), and highest PIC (0.283).

Significant differences in gene diversity, PIC, and MAF were found among the three subspecies (Supplementary Figure S2, Supplementary Table S4). The Tibetan semi-wild wheat accessions had the highest gene diversity, highest PIC, and lowest MAF compared with the other subspecies, with mean gene diversity of 0.37, mean PIC of 0.32, and mean MAF of 0.74. The Yunnan hulled wheat accessions had the lowest gene diversity (0.15) and PIC (0.13), and the highest MAF (0.90). The Xinjiang rice wheat accessions were intermediate with mean gene diversity of 0.26, mean PIC of 0.22, and mean MAF of 0.80.

### Population Structure and Linkage Disequilibrium

The filtered 38,490 polymorphic markers were used in the calculation of population structure and LD. On the basis of the Bayesian clustering, the 213 accessions were fell into two subpopulations by population structure analysis (Supplementary Figures S3, S4a). One subpopulation (Group 1) contained 141 accessions comprising 57 Tibetan semi-wild wheat, 75 Yunnan hulled wheat accessions, and nine Xinjiang rice wheat accessions. The other subpopulation (Group 2) contained 72 accessions comprising 60 Tibetan semi-wild wheat, three Yunnan hulled wheat, and nine Xinjiang rice wheat. Accessions of Tibetan semi-wild wheat and Xinjiang rice wheat were found in both groups, whereas almost all accessions of Yunnan hulled wheat were in Group 1. The results that the accessions of Tibetan semi-wild wheat and Xinjiang rice wheat classified in both groups are likely to be due to possible ancestral population structure. The subspecies of Tibetan semi-wild wheat and Xinjiang rice wheat are mainly divided by the growing regions and some morphological characteristics, but the origin of the subspecies might involve different ancestral populations.

The kinship matrix (*K*-matrix) was estimated to examine the genetic relationships among the 213 accessions. Hierarchical clustering of the *K*-matrix divided the 213 accessions into three clusters (Supplementary Figure S4b). Clusters 1, 2, and 3 contained 54, 86, and 73 accessions, respectively. Notably, almost all the accessions in Group 1 by Bayesian clustering were in Clusters 1 and 2 by hierarchical clustering, whereas all accessions in Group 2 were in Cluster 3.

**FIGURE 2:**
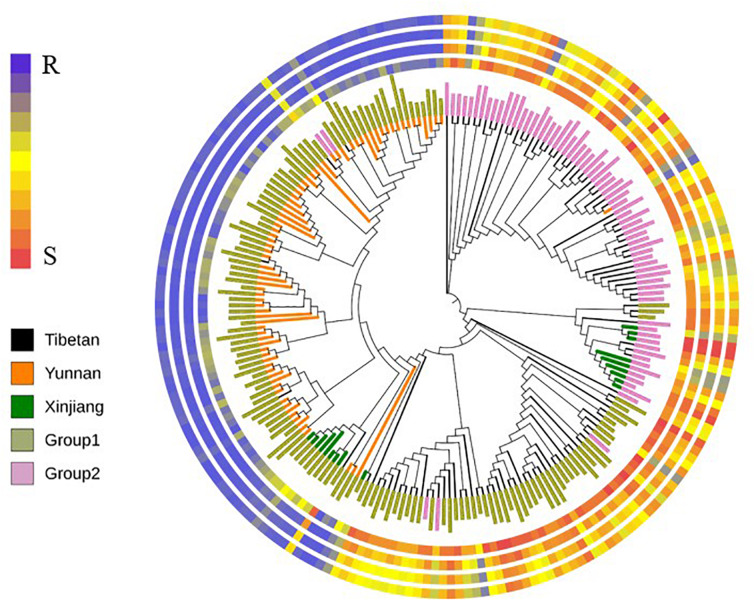
The neighbor-joining tree (NJ Tree) based on Nei’s distance. Colors of branches represented three subspecies. Tibetan represented Tibetan semi-wild wheat; Yunnan represented Yunnan hulled wheat; Xinjiang represented Xinjiang rice wheat. Colors of the names represented two subpopulations (Group 1 and Group 2). Four rings from inner to outer represented BLUP values of infection type (IT), final disease severity (DS), disease index and the area under disease progress curve (AUDPC), respectively.

The neighbor-joining tree (NJ Tree) showed similar distinct subpopulations by population structure analysis (Figure 2). On the basis of the BLUP values of IT, DS, DI, and AUDPC, the resistant accessions in Group 1 were predominantly Yunnan hulled wheat, whereas the susceptible accessions in Group 1 were predominantly Tibetan semi-wild wheat. Almost all accessions in Group 2 were susceptible.

The pairwise measure of LD was estimated based on the allele frequency correlations (*r*^2^) between a significant pair of SNP markers on the same chromosome with physical distances. The genome-wide half-decay distance was 2.57 Mb when the LD was 50% of its initial value (*r*^2^ = 0.65) (Supplementary Figure S5), and was considered to be the threshold for QTL identification in the GWAS analysis.

The AMOVA, fixation index (Fst), and Nm are provided in Table 2. The AMOVA revealed that 19% of the total variation was found among subpopulations, while, the rest of variation (81%) was within subpopulations. These results demonstrated that genetic differentiation among subpopulations was low and within subpopulations was high.

**TABLE 2:** Analysis of molecular variance among and within two subpopulations of 213 accessions in Chinese endemic wheat.

Source	*df*	SS	MS	Est. Var.	%	*P* value
Among Pops	1	99,652.597	99,652.597	1,001.787	19	0.010
Within Pops	211	877,408.811	4,158.336	4,158.336	81	0.010
Total	212	977,061.408		5,160.122	100	0.010
Fixation index (Fst)	0.194					
Nm	1.038					

### Genome-Wide Association Study Analysis of Resistance to Stripe Rust at the Adult Plant Stage

**FIGURE 3:**
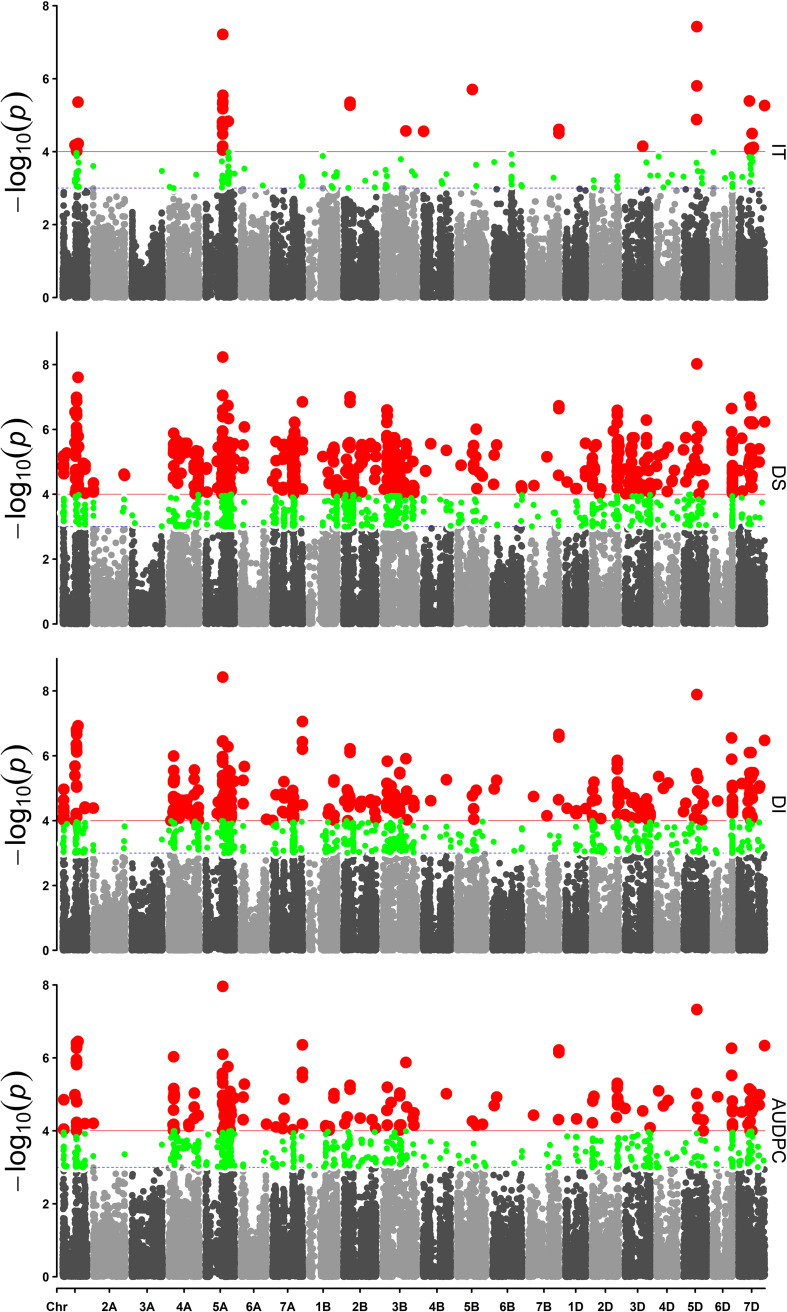
Manhattan plots of the markers associated with IT, DS, DI, and AUDPC in 19WJ. Red points above the red lines were considered as the significant associated locus where *P* < 0.0001. Green points between the red lines and blue dashed lines were markers with *P* < 0.001.

**FIGURE 4:**
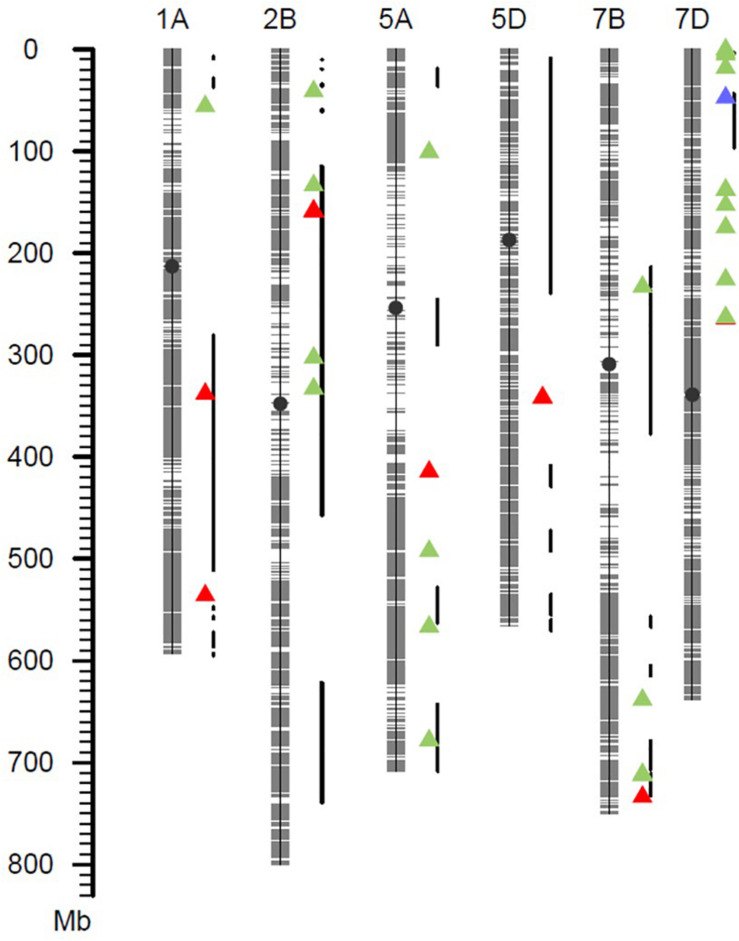
Exhibition of high-confidence QTLs and previously reported QTLs. Red triangles represented QTLs detected in this study. Green triangles represented QTLs detected in Chinese wheat landraces. Blue triangle represented *Yr18*. The thick black lines represented QTLs reported in the literature.

Based on a *Q* + *K* method for a mixed linear model with 38,490 SNP markers, association analysis for IT, DS, DI, and AUDPC was performed within each of the four field environments. The results were visualized as Manhattan plots (Figure 3). The QQ plots are shown in Supplementary Figure S6. A total of 11 SNP markers showed significant marker-wise associations with at least two of the four stripe rust resistance-associated traits (IT, DS, DI, and AUDPC) in three of the trials (Table 3). These markers were on chromosomes 1A, 2B, 5A, 5D, 7B, and 7D, and were divided into seven QTL regions on the basis of the half-decay distance. The phenotypic variation (*R*^2^) explained by these QTLs was 9.3–25.1%. Among them, QTL *QYr.sicau-7DS*, tagged by marker *AX-111118847*, showed the largest effect for stripe rust resistance (*R*^2^ = 0.26). QTL *QYr.sicau-2BS*, tagged by four significant markers, explained 11.3–24.7% of the phenotypic variation. Furthermore, compared with the loci reported previously on a consensus map and in the literature, two of the QTLs (*QYr.sicau-5AL* and *QYr.sicau-5DL*) were potentially novel QTLs because of their unique chromosomal locations (Figure 4). Three putative candidate genes of the two potentially novel QTLs were identified (Supplementary Table S5) by referencing the disease resistance-related protein family and signaling pathways. They were mainly genes that encode a UBX domain-containing protein, S-adenosyl-L-methionine-dependent methyltransferases superfamily protein, and zinc-finger homeodomain 1 protein.

**TABLE 3:** Summary of high-confidence associated markers.

QTL name	Marker	Chromosome	Position	Traits	Environments^a^	*R*^2b^	−*log*_10_(*P*)	FDR value (−*log*_10_(*q*))^c^
*QYr.sicau-1AL.1*	AX-110473633	1A	337993377	IT	18CZ, 19CZ1, BLUP	0.093–0.169	4.892–7.474	1.008–4.191
				DS	19CZ1, 19CZ2, 19WJ, BLUP	0.115–0.164	5.217–7.327	3.099–5.401
				DI	19CZ1, 19WJ, BLUP	0.108–0.122	4.789–5.312	2.500–3.467
*QYr.sicau-1AL.2*	AX-109409080	1A	535919449	IT	19CZ1, 19CZ2, BLUP	0.113–0.175	4.738–7.517	1.195–4.212
				DS	19CZ1, 19CZ2, 19WJ, BLUP	0.112–0.202	4.859–8.763	2.884–5.879
*QYr.sicau-2BS*	AX-109302093	2B	158642717	IT	19CZ1, 19CZ2, 19WJ, BLUP	0.122–0.226	5.357–9.719	1.700–5.436
				DS	19CZ1, 19CZ2, 19WJ, BLUP	0.161–0.247	7.004–10.649	3.199–6.367
				DI	19CZ1, 19CZ2, 19WJ, BLUP	0.113–0.205	4.737–8.635	2.017–5.182
				AUDPC	19CZ1, 19WJ, BLUP	0.122–0.206	5.251–8.714	1.857–5.710
	AX-109311497	2B	158776763	IT	19CZ1, 19CZ2, 19WJ, BLUP	0.121–0.223	5.274–9.558	1.804–5.673
				DS	19CZ1, 19CZ2, 19WJ, BLUP	0.159–0.244	6.836–10.269	3.366–6.463
				DI	19CZ1, 19WJ, BLUP	0.144–0.202	6.106–8.350	3.103–4.997
				AUDPC	19CZ1, 19WJ, BLUP	0.121–0.202	5.143–8.377	2.222–5.474
	AX-110644789	2B	159099539	IT	19CZ1, 19CZ2, 19WJ, BLUP	0.122–0.226	5.357–9.719	1.773–5.613
				DS	19CZ1, 19CZ2, 19WJ, BLUP	0.161–0.247	7.004–10.649	3.266–6.542
				DI	19CZ1, 19CZ2, 19WJ, BLUP	0.113–0.205	4.737–8.635	2.023–5.194
				AUDPC	19CZ1, 19WJ, BLUP	0.122–0.206	5.251–8.714	2.269–5.722
	AX-109323267	2B	159205262	IT	19CZ1, 19CZ2, 19WJ, BLUP	0.122–0.226	5.334–9.713	1.792–5.731
				DS	19CZ1, 19CZ2, 19WJ, BLUP	0.161–0.247	6.996–10.641	3.315–6.660
				DI	19CZ1, 19CZ2, 19WJ, BLUP	0.113–0.205	4.736–8.629	2.027–5.174
				AUDPC	19CZ1, 19WJ, BLUP	0.122–0.206	5.25–8.716	2.279–5.701
*QYr.sicau-5AL*	AX-111602206	5A	414417931	IT	19CZ2, 19WJ, BLUP	0.119–0.192	4.982–8.255	1.419–4.132
				DS	19CZ1, 19CZ2, 19WJ, BLUP	0.115–0.233	5.192–9.973	3.232–5.955
				DI	19CZ1, 19CZ2, 19WJ, BLUP	0.117–0.206	4.923–8.429	2.551–4.586
				AUDPC	19CZ1, 19WJ, BLUP	0.127–0.194	5.543–7.964	2.100–4.918
*QYr.sicau-5DL*	AX-109787548	5D	341595505	IT	19CZ1, 19CZ2, BLUP	0.106–0.158	4.689–7.000	1.419–4.132
				DS	19CZ1, 19CZ2, 19WJ, BLUP	0.139–0.205	6.091–8.993	3.232–5.955
				DI	19CZ1, 19WJ, BLUP	0.123–0.161	5.308–6.922	2.551–4.586
				AUDPC	19CZ1, 19WJ, BLUP	0.107–0.166	4.647–7.116	2.100–4.918
	AX-110894967	5D	342170812	IT	19CZ1, 19CZ2, BLUP	0.121–0.153	5.089–6.812	1.350–3.961
				DS	19CZ1, 19CZ2, 19WJ, BLUP	0.121–0.210	5.389–9.210	3.100–5.967
				DI	19CZ1, 19WJ, BLUP	0.113–0.178	4.915–7.565	2.444–4.667
*QYr.sicau-7BL*	AX-109582878	7B	733594988	IT	19CZ2, 19WJ, BLUP	0.106–0.164	4.609–7.159	1.223–4.238
				DS	19CZ1, 19CZ2, 19WJ, BLUP	0.109–0.196	4.857–8.386	3.374–6.095
				DI	19CZ1, 19WJ, BLUP	0.113–0.166	4.920–6.963	3.224–4.607
				AUDPC	19CZ1, 19WJ, BLUP	0.124–0.15	5.339–6.257	2.837–3.855
*QYr.sicau-7DS*	AX-111118847	7D	264233453	IT	19CZ1, 19CZ2, 19WJ, BLUP	0.124–0.26	5.390–10.558	1.584–5.974
				DS	19CZ1, 19CZ2, 19WJ, BLUP	0.162–0.251	6.994–10.732	3.366–6.148
				DI	19CZ1, 19CZ2, 19WJ, BLUP	0.143–0.226	5.847–9.288	1.740–4.705
				AUDPC	19CZ1, 19CZ2, 19WJ, BLUP	0.120–0.237	5.150–9.748	1.599–5.165

*IT, infection type; DS, final disease severity; DI, disease index; AUDPC, area under disease progress curve. ^a^18CZ, tests in Chongzhou at the year 2018; 19CZ1 and 19CZ2, tests in Chongzhou at the year 2019; 19WJ, tests in Wenjiang at the year 2019. ^b^Measurement of the percentage variation explained by each marker. ^c^q, false discovery rate (FDR) adjusted P-values.*

### Haplotype Analysis

**FIGURE 5:**
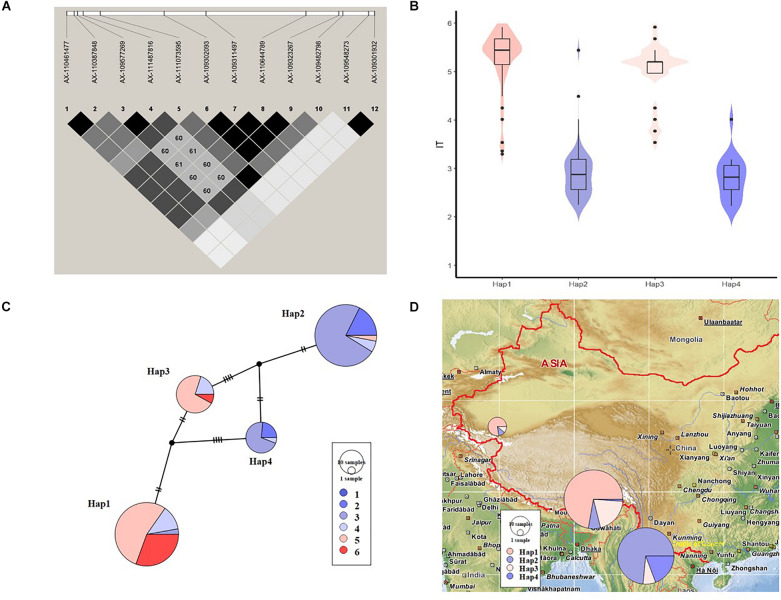
Haplotype analysis of a strongly associated QTL*QYr.sicau-2BS*. **(A)** Local linkage disequilibrium (LD) block for the 10 significant marker-trait associations based on the physical positions. **(B)** Violin plots and box plots illustrating the IT-BLUP values of accessions carrying different haplotype variants. **(C)** Resistance levels of accessions constituting four haplotype variants. Colors denoted mean IT-BLUP values (1–6) for those accessions. The size of the nodes and rings were proportional to the number of accessions. **(D)** Haplotype distributions of three subspecies on geographical map. The size of the rings indicated the number of accessions.

As a high-confidence QTL strongly associated with stripe-rust resistance detected in the field, *QYr.sicau-2BS* was selected for haplotype analysis. Ten markers formed a region of high LD (0.89 Mb) (Figure 5A), resulting in four common haplotypes (i.e., counts >5 in the whole population) in the association panel. Among these haplotypes, Hap2 and Hap4 showed mean IT-BLUP values of 2.94 and 2.82, respectively, which were significantly lower than those of Hap1 (5.53) and Hap3 (4.98) (Figures 5B,C and Table 4). The favorable haplotypes of Hap2 and Hap4 occurred in 68 and 17 of the accessions in the diversity panel, and both these haplotypes were found predominantly in the Yunnan hulled wheat accessions (Figure 5D).

**TABLE 4:** Summary of four haplotype variants.

Haplotypes	Total number	Subspecies			Response to *Pst*		Mean IT-BLUP value
		Tibetan^a^	Yunnan^b^	Xinjiang^c^	*R*^d^	*S*^e^	
Hap1	72	65	0	7	11	61	5.23
Hap2	68	6	61	1	66	2	2.94
Hap3	25	18	6	1	5	20	4.98
Hap4	17	1	16	0	17	0	2.82

*^a^Number of Tibetan semi-wild wheat accessions. ^b^Number of Yunnan hulled wheat accessions. ^c^Number of Xinjiang rice wheat accessions. ^d^Number of accessions resistant to the Pst. ^e^Number of accessions sensitive to the Pst.*

## Discussion

### Resistance Genes/QTLs Utilized in Chinese Wheat Breeding

To date, 83 formally designated genes (*Yr1*–*Yr83*) for stripe rust resistance (Li et al., 2020), 67 temporary nomenclature genes, and more than 330 QTLs have been identified on different chromosomes (Cheng et al., 2019). In China, only a small number of *Yr* genes/QTLs have been used in wheat breeding, including *Yr1*, *Yr5*, *Yr9*, *Yr10*, *Yr15*, *Yr17*, *Yr18*, and *Yr24/26*. The prevalence of a new *Pst* race CYR34 has gradually weakened most of the resistance genes or made them ineffective against stripe rust, including *Yr10* and *Yr24/Yr26*, which are widely used in breeding (Han and Kang, 2018). Only two resistance genes, *Yr5* and *Yr15*, are resistant to CYR34 (Liu et al., 2017), as well as the *Yr5*-virulence race “TSA-6” has been reported (Zhang et al., 2020). There is an ongoing need to search for new sources of genetic resistance to stripe rust due to the emergence of new races of the pathogen. Chinese endemic wheat has been historically underused in modern breeding programs, and thus are likely to represent new potential sources of novel resistance alleles.

### Different Responses to Stripe Rust Among the Three Chinese Endemic Wheat Subspecies

In this study, the stripe-rust resistance of 213 Chinese endemic wheat accessions, comprising Tibetan semi-wild wheat, Yunnan hulled wheat, and Xinjiang rice wheat, were identified preliminarily by phenotypic evaluation at the adult plant stage. Distinct responses to stripe rust were observed among the three subspecies in the field trials. Interestingly, almost all accessions of Tibetan semi-wild wheat were highly susceptible to stripe rust at the adult plant stage, whereas all accessions of Yunnan hulled wheat were highly resistant. Resistant and susceptible accessions were detected in Xinjiang rice wheat, which our results showed was intermediate between Tibetan semi-wild wheat and Yunnan hulled wheat. The differences in resistance might be caused by differences in selection pressure from *Pst* races. Stripe rust is widespread in the major wheat-growing areas of China, but is more prevalent in north-western and south-western regions, such as Yunnan (Zeng and Luo, 2006; Wan et al., 2007). Yunnan hulled wheat is grown mainly at altitudes of 1,500–2,500 m in the mountainous region of Yunnan Province (Dong et al., 1981), which is within the over-summering zone for stripe rust. Because of the relatively frequent epidemics of stripe rust in the wheat-growing region, Yunnan hulled wheat might be subject to strong selection pressure for resistance against *Pst* races. Conversely, Tibetan semi-wild wheat grows mainly on the Qinghai-Tibet Plateau at altitudes above 3,000 m (Yang et al., 1993). The relatively low temperature and rainfall in this area may limit stripe rust epidemics and result in low selection pressure for resistance to stripe rust (Chen, 2005). Xinjiang also is an over-summering area for stripe rust (Wan et al., 2007), so, for a long time, Xinjiang rice wheat might have been grown under moderate selection pressure for stripe rust resistance. Among these three subspecies, Yunnan hulled wheat is likely to be the most important resource for further exploration of stripe-rust resistance genes, and for resistance transfer and utilization in breeding.

Interestingly, almost all of the resistant genotypes were located in one clade on the phylogenetic tree, while susceptible genotypes were almost absent from this clade. To minimize the interference of the population structure, we have set a stringent threshold and more filter conditions (associated with at least two traits in at least three trials). As predicted, in different associated loci, the alleles associated with resistance were found in the resistant genotypes that were located out of the resistant clade, and alleles associated with susceptibility were found in the susceptible genotypes within the resistant clade. In addition, in haplotype analysis, accessions of two main haplotype variants (one was resistant and another was sensitive) were from both of the two subpopulations. The associated loci need to be verified by bi-parental populations in the further study.

### Physical Position-Based Comparison of *Pst*-Resistance *Yr* Genes and QTLs

Seven QTLs from 11 high-confidence loci located on six wheat chromosomes were significantly associated with resistance to *Pst* by GWAS analysis. Comparison of the seven QTLs and previously reported *Yr* genes and QTLs on the “Chinese Spring” physical map IWGSC RefSeq v1.0 are summarized in Figure 4.

Five of these QTLs may have been reported previously. *QYr.sicau-1AL.1* was detected for the IT, DS, or DI traits in at least three of the four environments. This QTL was located at about 337.99 Mb and overlapped with QTL *QYr.sun-1A* (Bariana et al., 2010). QTL *QYr.sicau-1AL.2* was located at about 535.92 Mb and was in close proximity to marker *wsnp_Ex_rep_c81556_76277906* (IWA5754), which is associated with stripe-rust resistance in synthetic hexaploid wheat (Zegeye et al., 2014). QTL *QYr.sicau-2BS* was tagged by four markers in a 0.57-Mb region and overlapped with QTL *QYr.ucw-2BS* (Cobo et al., 2018). QTL *QYr.sicau-7BL* with the associated marker *AX-109582878* was located at about 733.59 Mb and near QTLs *QTL-7BL.1* (Muleta et al., 2017a) and *QTL-7BL.3* (Muleta et al., 2017b). Marker *AX-1111188* linked to QTL *QYr.sicau-7DS* explained 26.0% of the phenotypic variation in the population. This location is distant from the hotspot region of *Yr18*, but close to marker *Xcfd14* linked to QTL *QIT.sicau-7D* (Long et al., 2019).

Two QTLs on chromosomes 5A and 5D did not correspond to any previously identified genes or QTLs for resistance to *Pst*, and thus are potentially novel loci. About 10 resistance QTLs have been mapped in the consensus map of chromosome 5A (Cheng et al., 2019), and most of them are in the distal region of chromosome 5AL in the physical map. QTL *QYr.sicau-5AL* identified in the present study was located at about 414.42 Mb in the upstream region of chromosome 5AL, far removed from the reported QTLs, which suggests it may be a novel QTL. QTL *QYr.sicau-5DL* tagged by markers *AX-109787548* and *AX-110894967* was located in the middle region of chromosome 5DL. To our knowledge, only a few of the reported QTLs are on chromosome 5D and none of them cover the location of *QYr.sicau-5DL* (Wang and Chen, 2017; Cheng et al., 2019), it also may be a novel QTL. These novel stripe rust resistance loci will be useful to diversify the current set of resistance genes that are used to control this devastating disease.

### Utilization of Chinese Endemic Wheat in Resistance Breeding

In the present study, stable stripe rust resistant germplasms were found among 213 Chinese endemic wheat accessions, and novel adult plant resistance loci for breeding and improvement of durable resistance in wheat were identified. Recent studies have indicated that the Chinese endemic wheat represents unique genetic resources with the potential to contribute novel alleles for broadening the current germplasm gene pools of breeding programs (Gu et al., 2015; Wang et al., 2019). However, a major disadvantage of Chinese endemic wheat is that they inevitably harbor genes that negatively impact agronomic traits, such as a brittle rachis, tough glume, and late maturation. Development of chromosome segment introgression lines (CSILs) may enable the transfer of one or a few chromosomal fragments of interest from donor parents to a receptor parent by crossing, backcrossing, and selfing (Eshed and Zamir, 1995). This method has been applied successfully to construct CSILs for improvement of agronomic traits using three accessions of Chinese endemic wheat (Gu et al., 2015). Future work will focus on utilization of CSILs and marker-assisted selection to facilitate transfer of the favorable QTLs from the Chinese endemic wheat to improve the durability of stripe rust resistance in wheat cultivars.

## Conclusion

It is necessary to explore novel stripe rust resistance loci from wild relatives of wheat for resistance breeding. Chinese endemic wheat, is a unique type of hexaploid wheat and is a promising source of novel loci for stripe rust resistance. We identified 89 accessions that displayed consistent resistance in four environments at the adult plant stage. Two of the seven QTLs strongly associated with resistance that were detected by GWAS analysis were novel QTLs. Haplotype variants revealed subspecies-specific inheritance of the stripe rust resistance-associated loci. Resistant loci in the Chinese endemic wheat germplasms could be used for wheat improvement. High-confidence SNP markers will aid in marker-assisted selection, and the resistant germplasm could be used as donor parents to construct CSILs in breeding for disease resistance in wheat.

## Data Availability Statement

The original contributions presented in the study are included in the article/Supplementary Material, further inquiries can be directed to the corresponding authors.

## Author Contributions

JL carried out the experiments, analyzed the data, and drafted the manuscript. YJ carried out the phenotypic evaluation, analyzed the genotypic data, and revised the manuscript. FY, LL, YqW, YW, HL, JW, QJ, HK, WL, and PQ carried out the phenotypic evaluation. JM, ZP, SD, and YmW participated in the field experiment and revised the manuscript. YZ participated in the design of the experiments. GC formulated the questions, designed and carried out the experiment, analyzed the data, and revised the manuscript. All authors read and approved the final version.

## Conflict of Interest

The authors declare that the research was conducted in the absence of any commercial or financial relationships that could be construed as a potential conflict of interest.

## Footnotes

1 https://megasoftware.net

2 https://itol.embl.de/

3 https://github.com/YinLiLin/R-CMplot

4 https://pan.baidu.com/s/1yePsGyPosZArxpwAo31xJw

5 https://wheat-urgi.versailles.inra.fr/Seq-Repository/Assemblies
