# Supplementary material for: Genome-Wide Association Study Reveals the Genetic Architecture of Stripe Rust Resistance at the Adult Plant Stage in Chinese Endemic Wheat
Source: Front Plant Sci. 2020 Jun 5;11:625. doi: 10.3389/fpls.2020.00625 (PMC7289992; doi:10.3389/fpls.2020.00625)
Supplement: Supplementary file 1 [file Data_Sheet_1.pdf]

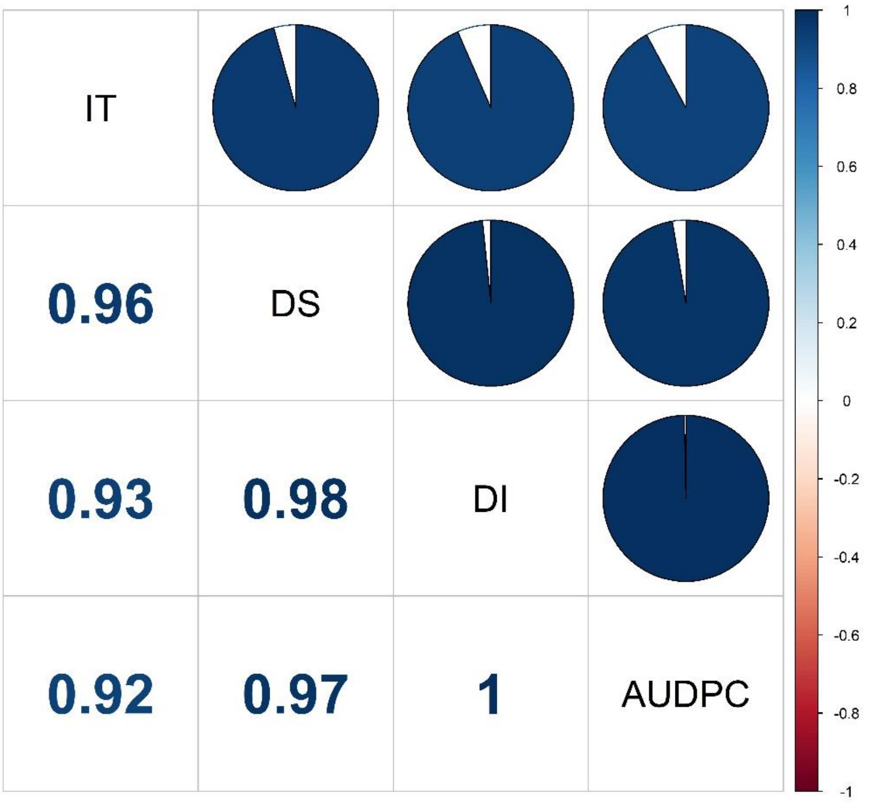

Fig. S1 The Pearson correlation analyses among infection type (IT), final disease severity (DS), disease index (DI), and the area under disease progress curve (AUDPC).

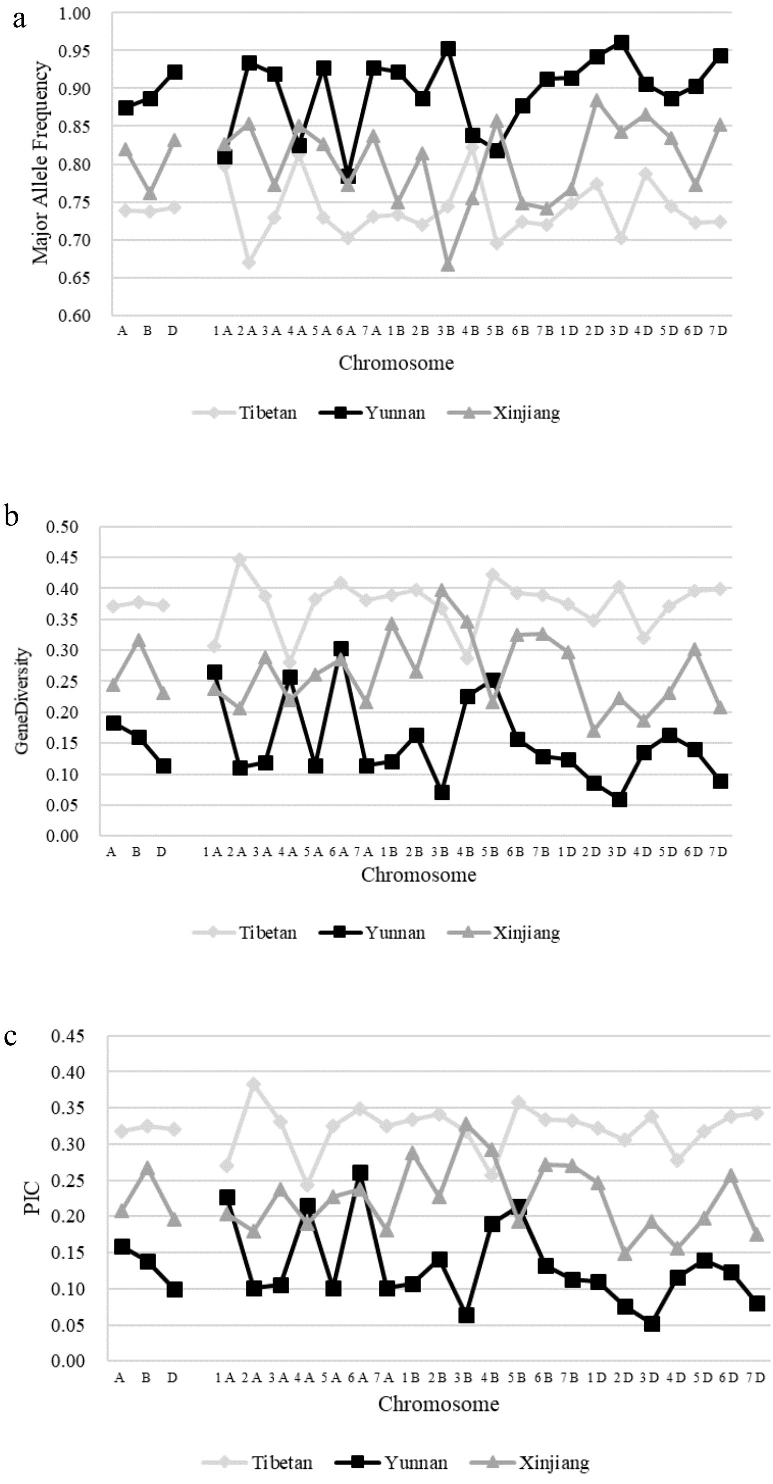

Fig. S2 Nei's major allele frequency (a), gene diversity (b) and polymorphism information content (PIC) (c) values of three subspecies. Tibetan, Tibetan semi-wild wheat; Yunnan, Yunnan hulled wheat; Xinjiang, Xinjiang rice wheat.

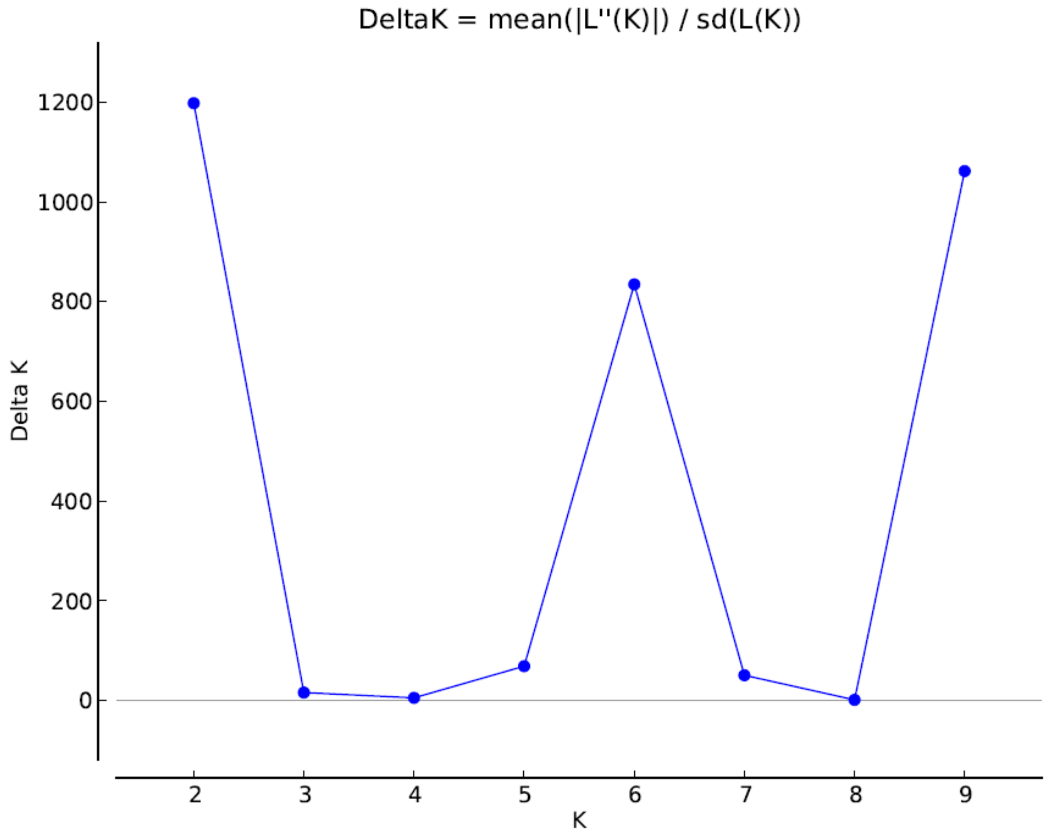

Fig. S3 Population structure analysis of 213 accessions by STRUCTURE HARVESTER, Delta *K* based on the rate of change of LnP(D) between successive *K*.

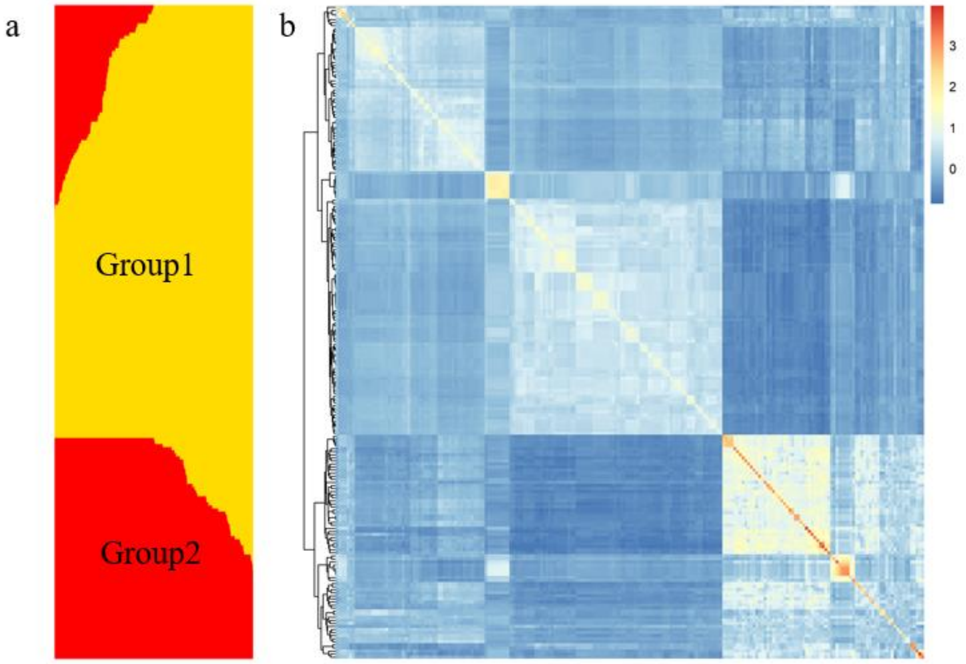

Fig. S4 Population structure of 213 Chinese endemic wheat accessions. (a) Two subpopulations based on the Bayesian clustering. Two colors stood for 2 different compositions. Different color areas of each line represented different proportions of composition. The subpopulation 1 (Group 1) mainly showed as yellow color. The subpopulation 2 (Group 2) mainly showed as red color. (b) Heat map relative with K-matrix.

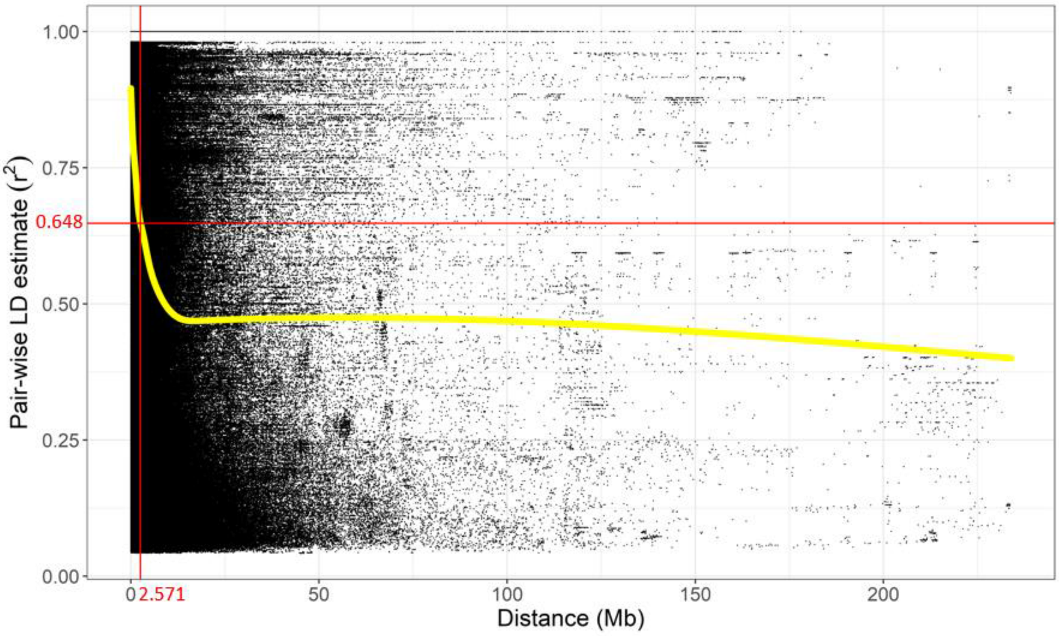

Fig. S5 Genome-wide average linkage disequilibrium (LD) decay over physical distances based on 38,490 SNP markers. The yellow curve represented the model fits to LD decay. The red lines represented that the half LD decay distance was 2.571 Mb when the LD declined to 50% (r^2 = 0.648) of its initial value. Mb, million base pairs LD decay based on SNP markers.

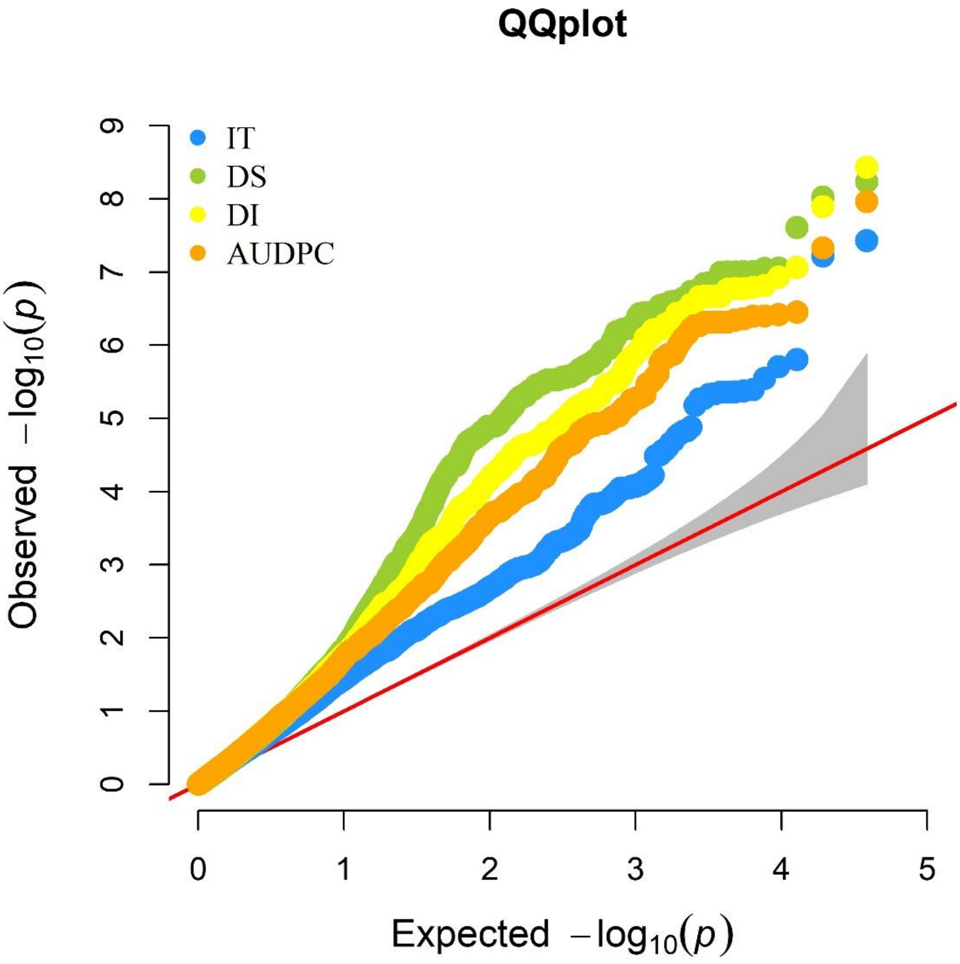

Fig. S6 The QQ plots of IT, DS, DI, and AUDPC in 19WJ.
